# Applying indirect open-circuit calorimetry to study energy expenditure in gnotobiotic mice harboring different human gut microbial communities

**DOI:** 10.1186/s40168-019-0769-4

**Published:** 2019-12-12

**Authors:** Ilia G. Halatchev, David O’Donnell, Matthew C. Hibberd, Jeffrey I. Gordon

**Affiliations:** 1 The Edison Family Center for Genome Sciences and Systems Biology, St. Louis, USA; 2 Center for Gut Microbiome and Nutrition Research, St. Louis, USA; 3 Department of Medicine, Washington University School of Medicine, St. Louis, MO 63110 USA

**Keywords:** Gnotobiotic mouse husbandry, Host phenotyping, Energy expenditure, Open-circuit indirect calorimetry, Body composition, Obesity, Human gut microbiota, Human donor gut bacterial culture collections

## Abstract

Given the increasing use of gnotobiotic mouse models for deciphering the effects of human microbial communities on host biology, there is a need to develop new methods for characterizing these animals while maintaining their isolation from environmental microbes. We describe a method for performing open-circuit indirect calorimetry on gnotobiotic mice colonized with gut microbial consortia obtained from different human donors. In this illustrative case, cultured collections of gut bacterial strains were obtained from obese and lean co-twins. The approach allows microbial contributions to host energy homeostasis to be characterized.

## Background

Studies of wild-type or genetically engineered mice maintained under germ-free conditions, or colonized with microbes derived from healthy or diseased humans (or other species), have been instrumental in advancing understanding of the assembly, dynamic operations, functional properties, and biological effects of microbial communities. Gnotobiotic animal models have and will continue to play a key role in advancing the field of human microbiome research, both in terms of developing new experimental and computational approaches that reveal basic mechanisms underlying microbial-microbial and microbial-host interactions, and in developing new approaches for microbiome-based therapeutics. As such, there is a pressing need to develop new and/or improved methods for delineating the physiologic, metabolic, immunologic, neurologic, and other features of these animals over time, within the confines of gnotobiotic isolators, as a function of their colonization status and various environmental perturbations [1].

Development of obesity is the result of a net positive balance between energy intake and energy expenditure. This balance is governed by “intrinsic factors,” including a variety of hormones, neurocircuits, and host genetics that regulate food consumption and energy expenditure, as well as by “extrinsic factors,” such as living environment and the composition of consumed food. The contributions of the gut microbiota to obesity and its accompanying metabolic dysfunctions are being investigated in a number of human populations and in model organisms. The latter includes studies of germ-free animals colonized with intact uncultured gut microbial communities harvested from obese or lean humans or cultured components of these communities. Recipient animals are fed a variety of experimental diets, including those designed to represent those consumed in Westernized societies. The results of such studies have provided preclinical evidence that the gut microbiota is a factor that regulates adiposity, contributes to the metabolic abnormalities associated with obesity, and influences the rate of weight regain following weight loss induced by dieting [2–5].

Open-circuit, indirect calorimetry is a non-invasive method for studying energy expenditure [6]. Developing methods for adapting this procedure to gnotobiotic isolators containing mice colonized with gut microbes from various human donors, and fed representative human diets, provides a means for characterizing how gut community members contribute to host energy homeostasis and metabolism. A previous study examined mice that had been mono-colonized in a gnotobiotic isolator with wild-type or mutant strains of a human gut bacterial species (*Bacteroides thetaiotaomicron*) [7]. Just prior to sacrifice, as part of an analysis of the effects of colonization, animals were transferred to a calorimetry apparatus located outside of the isolator. Here we describe an apparatus and procedure for calorimetry of mice maintained for sustained periods of time under gnotobiotic conditions and directly test the hypothesis that different consortia of human gut bacteria introduced into these mice fed a representative low saturated fat, high-fiber diet consumed in the USA can produce a measurable difference in host energy expenditure.

## Results

**Fig. 1:**
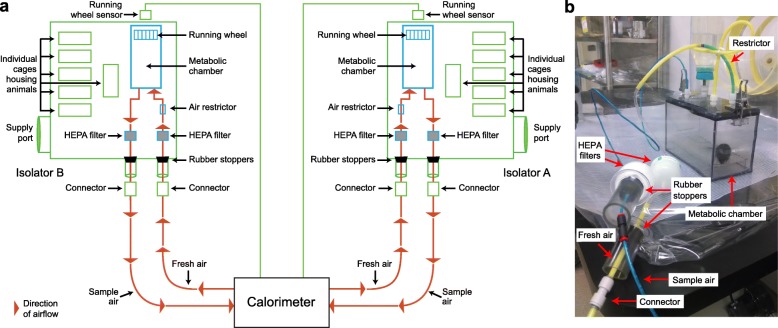
Indirect open-circuit calorimetry in gnotobiotic isolators. **a** Schematic drawing of the experimental apparatus. Two isolators are shown: isolator A contains animals colonized with bacterial consortium A from the lean co-twin; isolator B contains animals harboring bacterial consortium B from the obese co-twin. **b** Photograph of flexible film gnotobiotic isolator containing a metabolic chamber and devices used to connect the chamber to the exterior calorimeter

**Fig. 2:**
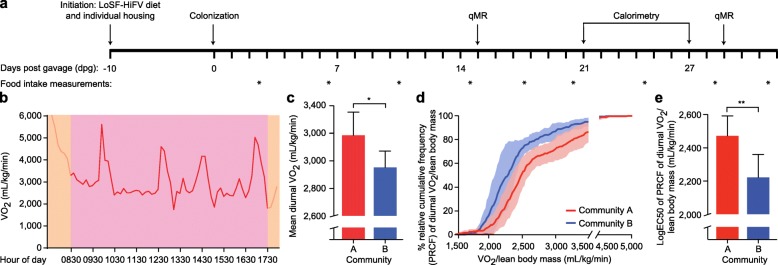
Comparison of the effects of two different consortia of cultured human gut bacterial strains on energy expenditure. **a** Experimental protocol. Mice underwent qMR measurements, indirect open-circuit calorimetry, and quantitation of food intake at the time points indicated. **b** Example of diurnal indirect open-circuit calorimetry of a mouse colonized with consortium A placed in the metabolic chamber at 0600 h with free access to water but not food. Red tracing indicates the measurements used to calculate mean diurnal VO_2_. Orange denotes periods excluded from the analysis when the mouse was initially placed in the metabolic chamber and when the chamber was open for introduction of food at 1730 h. **c** Mean diurnal VO_2_ in mice colonized with bacterial consortium A from the lean co-twin compared to bacterial consortium B from the obese co-twin (data derived from measurements made from six animals/treatment group from dpg 21 to 27; mean values ± SD are plotted). **d** Percent relative cumulative frequency (PRCF) of diurnal VO_2_ in mice colonized with consortium A compared to mice colonized with consortium B. Solid lines represent mean values with the shaded areas indicating the standard deviation. **e** LogEC50 of PRCF of diurnal VO_2_ in animals containing consortium A and consortium B. *P < 0.05; **P < 0.01 (Student’s *t* test). Abbreviations; qMR, quantitative magnetic resonance; LoSF-HiFV, low saturated fat, high fruits and vegetables diet

Figure 1 and the “Methods” section describe how flexible plastic film gnotobiotic isolators were adapted for indirect open-circuit calorimetry experiments. Figure 2a outlines the design of an illustrative experiment. Twelve-week-old germ-free male C57BL/6 J mice were individually housed in two gnotobiotic isolators using a protocol detailed in the “Methods” section. At the time of initiation of the experiment, animals were begun on a diet representing the lower third of consumption of saturated fats and the upper third of consumption of fruits and vegetables reported in 1-day recalls by participants in the U.S. National Health and Nutrition Examination Survey (NHANES). This diet, abbreviated LoSF-HiFV, was constructed using commercially available human foods and was cooked prior to sterilization by gamma irradiation (for details see ref. 2). We had previously shown that this diet supports the colonization of germ-free mice with defined consortia of phylogenetically diverse cultured gut bacterial strains recovered from the fecal microbiota of lean and obese twins [2, 8].

Two groups of animals were studied (*n* = 6/group), each in their own gnotobiotic isolator. Each isolator contained a single metabolic chamber. Two weeks prior to colonization, the germ-free mice were introduced into their isolator and maintained under a strictly controlled 12:12 L:D cycle with lights on between 0600 h and 1800 h (diurnal period) and lights off between 1800 h and 0600 h (nocturnal period). Four days after introduction into their isolator, animals were singly caged (6 cages/isolator). Animals were given the LoSF-HiFV human diet ad lib for 10 days prior to gavage (Fig. 2a). During this period, the germ-free status of mice was verified by culture-based analysis of serially collected fecal samples. Ten days after initiation of single-housing, animals were colonized, by oral gavage, with a consortium of bacterial strains cultured from a fecal microbiota sample obtained from one of two human donors. The donors were adult female dizygotic twins where the two siblings were stably discordant for obesity [2]. Consortium A from the lean co-twin (body mass index, 23 kg/m^2^) was administered to mice residing in one gnotobiotic isolator, while consortium B from the obese co-twin (body mass index, 32 kg/m^2^) was gavaged into mice in the other gnotobiotic isolator. The bacterial communities implanted in the two groups of recipient animals had distinct compositions (see Table 1 for their membership as defined by sequencing 16S rDNA amplicons generated from fecal samples collected at 18- and 32-days post-gavage (dpg)).

**Table 1:** V4-16S rDNA analysis of the percent relative abundances of members of consortium A and consortium B in the fecal microbiota of recipient gnotobiotic mice

ASV	Family	Genus	Species	Consortium A		Consortium B	
				dpg 18	dpg 32	dpg 18	dpg 32
				Percent relative abundance (± standard deviation)			
ASV1	Ruminococcaceae	*Subdoligranulum*		1.7 ± 0.7	2.1 ± 0.7	3.3 ± 0.5	3.7 ± 0.9
ASV2	Verrucomicrobiaceae	*Akkermansia*		2 ± 0.7	2.1 ± 1.1	0 ± 0	0 ± 0
ASV3	Rikenellaceae	*Alistipes*	*shahii*	0.2 ± 0.1	0.2 ± 0.1	0 ± 0	0 ± 0
ASV4	Bacteroidaceae	*Bacteroides*		9.7 ± 5.4	10.4 ± 1.5	0 ± 0	0 ± 0
ASV5	Bacteroidaceae	*Bacteroides*	*massiliensis*	2.9 ± 1.3	1.4 ± 0.6	3.6 ± 8.8	3.4 ± 8.3
ASV6	Bacteroidaceae	*Bacteroides*	*uniformis*	6.7 ± 4.5	5 ± 1.9	0 ± 0	0 ± 0
ASV7	Bacteroidaceae	*Bacteroides*		6.6 ± 3.9	6.5 ± 3.8	0 ± 0	0 ± 0
ASV8	Bacteroidaceae	*Bacteroides*	*caccae*	5.4 ± 3.2	5.3 ± 3.6	0 ± 0	0 ± 0
ASV9	Bacteroidaceae	*Bacteroides*		6.9 ± 5.7	6.4 ± 3.8	0 ± 0	0 ± 0
ASV10	Bacteroidaceae	*Bacteroides*	*ovatus*	5.5 ± 2.6	4.1 ± 1.6	8.8 ± 6.9	9.2 ± 4.6
ASV11	Bacteroidaceae	*Bacteroides*	*ovatus*	8.5 ± 2.9	7.2 ± 3.2	14.1 ± 11	14.1 ± 7.1
ASV12	Bacteroidaceae	*Bacteroides*	*ovatus*	2.2 ± 0.8	2.1 ± 1.1	0 ± 0	0 ± 0
ASV13	Porphyromonadaceae	*Parabacteroides*		0 ± 0	0 ± 0	3.5 ± 1.6	2.8 ± 1.6
ASV14	Porphyromonadaceae	*Parabacteroides*		0 ± 0	0 ± 0	3.5 ± 1.8	3 ± 1.5
ASV15	Porphyromonadaceae	*Parabacteroides*	*distasonis*	4 ± 1.9	4.8 ± 1.7	11.1 ± 5.7	9.6 ± 5.3
ASV16	Porphyromonadaceae	*Parabacteroides*	*distasonis*	1.3 ± 2.2	0.9 ± 1.3	8.2 ± 4.2	7.3 ± 4.2
ASV17	Porphyromonadaceae	*Parabacteroides*	*merdae*	3.3 ± 1.2	3.9 ± 0.8	0 ± 0	0 ± 0
ASV18	Rikenellaceae	*Alistipes*	*putredinis*	2 ± 0.2	1.7 ± 0.5	0 ± 0	0 ± 0
ASV19	Enterobacteriaceae	*Escherichia*		0.7 ± 0.5	1.6 ± 0.7	0 ± 0	0 ± 0
ASV20	Porphyromonadaceae	*Butyricimonas*	*paravirosa*	0.4 ± 0.3	0.4 ± 0.2	0 ± 0	0 ± 0
ASV21	Ruminococcaceae	*Butyricicoccus*		0.5 ± 0.2	0.3 ± 0.1	0 ± 0	0 ± 0
ASV22	Catabacteriaceae	*Catabacter*	*hongkongensis*	0.2 ± 0.1	0.2 ± 0.1	0 ± 0	0 ± 0
ASV23	Ruminococcaceae	*Anaerotruncus*	*colihominis*	0.2 ± 0.1	0.2 ± 0.1	0 ± 0	0 ± 0
ASV24	Coriobacteriaceae	*Eggerthella*	*lenta*	0 ± 0	0 ± 0	0.1 ± 0.1	0.2 ± 0.1
ASV25	Ruminococcaceae	*Ruminococcus*	*bicirculans*	0.2 ± 0.1	0.3 ± 0.1	0 ± 0	0 ± 0
ASV26	Ruminococcaceae	*Clostridium*		0.1 ± 0.1	0.1 ± 0.1	0 ± 0	0 ± 0
ASV27	Eubacteriaceae	*Eubacterium*		3.1 ± 2	3.4 ± 1	0 ± 0	0 ± 0
ASV28	Lachnospiraceae	*Clostridium*	*lactatifermentans*	0 ± 0	0 ± 0	1.4 ± 0.4	1.3 ± 0.5
ASV29	Lachnospiraceae	*Anaerostipes*	*caccae*	0 ± 0	0 ± 0	1.1 ± 0.4	1.2 ± 0.3
ASV30	Lachnospiraceae	*Blautia*		0 ± 0	0 ± 0	16.6 ± 6.9	18.6 ± 8.6
ASV31	Lachnospiraceae	*Blautia*		0 ± 0	0 ± 0	2.5 ± 2.8	3 ± 2.9
ASV32	Lachnospiraceae	*Blautia*		0 ± 0	0 ± 0	3.1 ± 3.5	3.7 ± 3.5
ASV33	Lachnospiraceae			0 ± 0	0 ± 0	0.2 ± 0.1	0.2 ± 0.1
ASV34	Lachnospiraceae	*Clostridium*		0 ± 0	0 ± 0	1.5 ± 1	1.3 ± 0.9
ASV35	Lachnospiraceae	*Clostridium*		0 ± 0	0 ± 0	7 ± 3.6	6.3 ± 3
ASV36	Lachnospiraceae	*Clostridium*		2.8 ± 1.5	3.4 ± 0.5	1.7 ± 0.4	1.8 ± 0.5
ASV37	Lachnospiraceae	*Clostridium*	*aldenense*	0.8 ± 0.4	0.9 ± 0.2	0.3 ± 0.3	0.4 ± 0.2
ASV38	Lachnospiraceae	*Hungatella*		4.1 ± 1.7	4.5 ± 1.2	0 ± 0	0 ± 0
ASV39	Lachnospiraceae	*Clostridium*		0.4 ± 0.4	0.5 ± 0.1	0 ± 0	0 ± 0
ASV40	Coriobacteriaceae	*Collinsella*	*aerofaciens*	1.3 ± 0.6	1.8 ± 0.5	0 ± 0	0 ± 0
ASV41	Peptostreptococcaceae	*Terrisporobacter*		0.3 ± 0.3	0.8 ± 0.7	0 ± 0	0 ± 0
ASV42	Bifidobacteriaceae	*Bifidobacterium*		1.1 ± 1.3	1.2 ± 1.1	0 ± 0	0 ± 0
ASV43	Bifidobacteriaceae	*Bifidobacterium*		1.2 ± 2.1	0.4 ± 0.6	0.2 ± 0.1	0.4 ± 0.4
ASV44	Sutterellaceae	*Parasutterella*	*excrementihominis*	5.2 ± 1.4	6 ± 1.4	0 ± 0	0 ± 0
ASV45	Sutterellaceae	*Parasutterella*		0.8 ± 0.2	0.9 ± 0.3	0 ± 0	0 ± 0
ASV46	Ruminococcaceae	*Clostridium*		0.3 ± 0.2	0.4 ± 0.1	0 ± 0	0 ± 0
ASV47	Erysipelotrichaceae			0.7 ± 0.4	0.9 ± 0.2	0.7 ± 0.2	0.9 ± 0.2
ASV48	Ruminococcaceae	*Flavonifractor*		0.3 ± 0.1	0.4 ± 0.1	0 ± 0	0 ± 0
ASV49	Ruminococcaceae	*Flavonifractor*	*plautii*	1.4 ± 0.2	1.5 ± 0.3	2.3 ± 0.6	2.2 ± 0.3
ASV50	Ruminococcaceae	*Acetanaerobacterium*		1.7 ± 0.7	1.5 ± 0.4	0 ± 0	0 ± 0
ASV51	Ruminococcaceae	*Clostridium*		1 ± 0.4	1.1 ± 0.3	0 ± 0	0 ± 0
ASV52	Enterococcaceae	*Enterococcus*		0 ± 0	0 ± 0	0.6 ± 0.2	0.8 ± 0.6
ASV53	Enterococcaceae	*Enterococcus*		0.6 ± 0.3	1.3 ± 1.9	0.8 ± 0.9	1.6 ± 1.1
ASV54	Ruminococcaceae	*Pseudoflavonifractor*		0.3 ± 0.2	0.3 ± 0.1	0 ± 0	0 ± 0
ASV55	Lachnospiraceae			0.9 ± 0.4	1.1 ± 0.2	0 ± 0	0 ± 0
ASV56	Lachnospiraceae	*Ruminococcus*		0.6 ± 0.3	0.7 ± 0.2	0 ± 0	0 ± 0
ASV57	Lachnospiraceae	*Ruminococcus*		0 ± 0	0 ± 0	3.5 ± 1	3 ± 1.2

Following gavage, mice were habituated to feeding from 1800–0600 h for the duration of the experiment. Animals were given access to food ad libitum only during the nocturnal period. The “cycle” of calorimetry measurement lasted for 6 days beginning at 21 dpg, with each animal in a given group sequentially placed in their gnotobiotic isolator’s metabolic chamber for 24 h. The chamber was connected to the calorimeter in a manner, described in Fig. 1a and the “Methods” section, that prevented exposure of the mice to sources of microbes outside of their isolator (including contamination with microbes harbored by the other treatment group). Diurnal and nocturnal calorimetry data were collected throughout this 24-h period (Fig. 2b, “Methods” section), and then normalized to total body weight (measured using scales located within the isolator) and lean body mass (LBM; defined by quantitative magnetic resonance (qMR), see “Methods” section).

Data were analyzed using (i) the mean of diurnal and nocturnal VO_2_ (all measurements made for each animal during the respective time periods were summed and normalized to the number of measurements), (ii) the sum of diurnal and nocturnal VO_2_ by percent relative cumulative frequency (PRCF), where the frequency of individual VO_2_ measurements was summed to produce a curvilinear distribution of VO_2_ [9] from which a logEC50 was calculated, and (iii) the type of energy source utilized (carbohydrate versus lipid) which was evaluated by examining the mean of diurnal and nocturnal respiratory exchange ratio (RER; all RER measurements made for each animal during the respective time period were summed and normalized to the number of measurements).

Figure 2c shows that mean *diurnal* (resting) VO_2_ was significantly higher in mice harboring consortium A from the lean co-twin compared to consortium B from the obese co-twin, as defined by the mean VO_2_ of the total number of measurements normalized to total body weight. While differences in *nocturnal* VO_2_ normalized to total body weight did not achieve statistical significance, animals containing consortium A trended toward higher values (4827.52 ± 512.6 versus 4307.66 ± 340.8 mL/kg/min (mean ± SD) for mice colonized with consortium A and consortium B, respectively; *p* = 0.065 (Student’s *t* test)).

The VO_2_ of metabolically active LBM is known to be the major contributor to energy expenditure [10]. Compared to mice harboring consortium B, animals colonized with consortium A had significantly higher VO_2_ per LBM during the diurnal period, with their PRCF curve shifted to the right (Fig. 2d) and higher logEC50 (Fig. 2e). This was also true during the nocturnal period (logEC50 normalized to LBM and the number of nocturnal measurements, 3996.7 ± 442.8 versus 3376.7 ± 374.3 mL/kg/min (mean ± SD) for animals harboring consortium A and consortium B, respectively; *p* = 0.026 (Student’s *t* test)). There were no significant differences in spontaneous nocturnal exercise in the metabolic chamber (71.9 ± 51.8 versus 49.9 ± 23.8 (mean ± SD) running wheel revolutions/night for animals harboring the A and B consortia, respectively; *p* = 0.37 (Student’s *t* test)).

While there were no significant differences in LBM between the two groups of mice when measured by qMR at 15 and 29 dpg, fat mass was significantly lower in mice harboring consortium A (i.e., those with a higher VO_2_). There was no statistically significant difference in total body weight, normalized to starting weight, between the two groups of mice at these time points (Table 2). Food intake was not significantly different between the two groups of animals at any of the nine time points surveyed (Table 2).

**Table 2:** Analysis of body composition, food intake, and weight in gnotobiotic mice colonized with consortium A or consortium B

Body composition (defined by qMR)			
dpg	Consortium A	Consortium B	*p* value*
	Mean ± SD	Mean ± SD	
Fat mass (grams)			
15	4.10 ± 0.37	4.90 ± 0.38	*p* < 0.05
29	4.21 ± 0.52	5.23 ± 0.69	*p* < 0.01
Lean body mass (grams)			
15	21.58 ± 1.27	20.82 ± 1.43	ns
29	21.67 ± 0.93	21.65 ± 1.44	ns
Food intake (grams)			
4	4.22 ± 0.97	4.48 ± 2.16	ns
7	3.63 ± 0.52	2.77 ± 0.41	ns
11	2.90 ± 0.55	2.51 ± 0.53	ns
15	3.00 ± 0.46	3.00 ± 0.39	ns
18	2.88 ± 0.48	2.73 ± 0.31	ns
21	2.88 ± 0.23	2.87 ± 0.35	ns
25	3.38 ± 0.62	3.08 ± 0.73	ns
29	3.42 ± 0.90	3.25 ± 0.47	ns
32	3.15 ± 0.37	2.88 ± 0.47	ns
Weight (normalized to starting weight)			
4	1.02 ± 0.04 (6)	1.00 ± 0.03 (6)	ns
7	1.02 ± 0.04 (6)	1.00 ± 0.03 (6)	ns
11	1.02 ± 0.03 (6)	1.01 ± 0.04 (6)	ns
15	1.01 ± 0.03 (6)	1.01 ± 0.04 (6)	ns
18	1.01 ± 0.04 (6)	1.02 ± 0.03 (6)	ns
21	1.02 ± 0.05 (6)	1.02 ± 0.04 (6)	ns
25	1.04 ± 0.05 (6)	1.03 ± 0.04 (6)	ns
29	1.02 ± 0.04 (6)	1.05 ± 0.04 (6)	ns
32	1.03 ± 0.06 (6)	1.06 ± 0.04 (6)	ns

*2-way ANOVA (*n* = 6 animals/group)

Measurements of RER disclosed that mice transplanted with either community used the same substrates for energy; predominantly fat during the diurnal period when they were resting and food was not available (0.73 ± 0.002 versus 0.73 ± 0.006 (mean ± SD) for mice with consortium A and consortium B, respectively; *p* = 0.51 (Student’s *t* test)), and carbohydrates during the nocturnal period when they were active and food was provided ad lib (0.97 ± 0.01 versus 0.97 ± 0.03 (mean ± SD) for groups with consortium A and consortium B, respectfully; *p* = 0.72 (Student’s *t* test)).

## Discussion

We describe a method for performing indirect open-circuit calorimetry within gnotobiotic isolators housing mice. This method allows assessment of the effects of different gut microbial communities from different human donors, in various diet contexts, on energy intake and expenditure, types of fuels utilized, and body composition.

Our protocol was designed to acclimate mice to their gnotobiotic environment in order to reduce stress and improve the reliability of the calorimetric measurements. Specifically, animals were brought into the gnotobiotic isolator 2 weeks prior to initiation of experiments, individually housed for 10 days prior to gavage, and given the LoSF-HiFV human diet ad lib for 10 days. Following gavage, they were habituated to feeding from 1800–0600 h for the duration of the experiment. Providing food ad libitum only during the 12-h nocturnal period does not simulate the normal feeding behavior, as many strains of mice normally consume a substantial portion (approximately 25%) of their food during the day. However, we restricted feeding to this in a 12-h interval in an effort to more accurately assess basal metabolic activity by reducing sporadic scavenging for food and by promoting resting/sleeping during the diurnal period. We found that a single 24-h period of pre-exposure to the metabolic chamber was sufficient for mice to acclimate to this environment; during subsequent calorimetry measurements, all animals would go to sleep within 30 min after placement in the chamber, with only intermittent episodes of waking (see the representative VO_2_ consumption profile presented in Fig. 2b). The calorimeter can be equipped to measure multiple channels such that multiple metabolic cages can be monitored simultaneously (i.e., in parallel). Alternatively, a single channel can be used to monitor one metabolic cage at a time, i.e., multiple animals can be characterized in a series of successive measurements (the approach used in this report).

The value of using culture collections from donors with phenotypes of interest is that if the collections have been clonally arrayed in multi-well plates with one strain/well, the composition of the consortium used to colonize animals can be systematically manipulated.

Our experimental design allows for future studies where repeated rounds of calorimetry are performed, serially or in parallel, of male and/or female mice representing different genetic backgrounds (inbred or engineered), ages, or sex, colonized with unmanipulated or manipulated defined communities of cultured microbes (e.g., “leave one or more organisms out” prior to gavage of a consortium) or with intact uncultured microbiota. Our experimental setup also allows for repeated measurements of controls that are maintained as germ-free throughout the experiment—something that was not done for the current study which focused on whether there were appreciable differences in VO_2_ consumption between animals harboring two distinct model human gut communities. Adding a germ-free arm would further advance understanding of the contributions of a microbial community and its constituents to energy homeostasis. Together, these types of calorimetric experiments, performed using the experimental apparatus described in the present report, would help frame hypothesis-based dissection of the mechanisms by which gut microbial communities produce their effects on various features of host metabolism [11].

## Methods

### Bacterial culture collections

Bacterial culture collections were generated from human fecal samples that had been collected during a previously described study [2] and subsequently maintained at – 80 °C. This previous study was approved by the Washington University Human Research Protection Office [2].

### Defining microbial community composition in gnotobiotic mice

The germ-free status of mice prior to colonization was defined by culturing fecal pellets in brain heart infusion (BHI) broth, nutrient broth, and Sabouraud-dextran broth (Difco) for 1 week at 37 °C under aerobic conditions, and in Tryptic Soy broth (Difco) under anaerobic conditions (atmosphere; 75% N_2_, 20% CO_2_, and 5% H_2_). In a series of control experiments, we applied the same culture-based method to show that our protocol for transferring germ-free animals to the qMR machine and back to the isolator or to metabolic cages connected to the calorimeter did not result in contamination.

Methods used for isolation of DNA from frozen fecal samples obtained from animals colonized with the cultured bacterial consortia, generation of V4-16S rDNA amplicons, and sequencing these amplicons (250 nucleotide paired-end reads; Illumina MiSeq instrument) are described in a previous publication [12]. Amplicon Sequence Variants (ASVs) were identified and quantified using DADA2 [13] and taxonomy was assigned using the DADA2 “assignTaxonomy” tool and the Ribosomal Database Project Training Set v16. The resulting ASV table was rarefied to even depth (*n* = 18,900 reads/sample) and filtered to include only ASVs with ≥ 0.1% relative abundance in at least 30% of analyzed fecal samples.

### Performing open-circuit indirect calorimetry in gnotobiotic isolators

Figure 1 describes how flexible plastic film gnotobiotic isolators (Class Biological Clean, Madison, WI) were adapted for indirect open-circuit calorimetry experiments. Two 3-cm ports were placed on the side of the isolator for the metabolic cage contained within the isolator. One port allowed “fresh air” (room air) to flow at a constant rate from the calorimeter (Oxymax Flow Max 210; Columbus Instruments) into a metabolic chamber, while the other port allowed “sample air” exiting the metabolic chamber to return to the calorimeter for measurement of O_2_ and CO_2_ content.

Fresh air coming from the calorimeter passed through a disposable, high-efficiency particulate air (HEPA)-Cap disposable in-line filter (Whatman; catalog number 6702-3600) that removes particulate material > 0.3 μm in diameter, then a 5.35-mm internal diameter polyethylene plastic tube, followed by a smaller 3.1-mm internal diameter tube that functions as an air restrictor and enters the metabolic chamber. An exhaust vent from each metabolic chamber carried “sample air” through 3.1-mm internal diameter tubing that exited the isolator after passing through another HEPA filter and was “returned” to the calorimeter for measurement of O_2_ consumption and CO_2_ production.

All equipment destined to reside inside the isolator (metabolic cages and flooring, connecting tubes, HEPA filters, an exercise wheel, and digital scale) were placed inside instant sealing sterilization pouches (Fischer), and the bags were subjected to ethylene oxide sterilization (3 M Steri-Vac Sterilizer/Aerator Model 8XL) using conditions recommended by the manufacturer. Pouches were subsequently introduced into the main supply transfer port of the gnotobiotic isolator, the port was almost completely closed, and the outside surfaces of the pouches were sterilized within the port by introducing chlorine dioxide gas (one part Clidox-S base: one part Clidox-S activator: three parts water), followed by full port closure and a 5-h period of sterilization. The interior plastic barrier separating the transfer port from the isolator was then removed, the equipment was passed into the isolator, and the connections between the metabolic cages and calorimeter were established to maintain sterility.

Two weeks prior to gavage, mice were transferred from breeder isolators along with bottled sterile water, sterilized food, sterilized bedding (Aspen chips), and standard polycarbonate mouse cages (Allentown). Transfer was achieved by attaching a transfer sleeve to the outer opening of the transfer port and sterilizing the interior of the sleeve with chlorine dioxide gas as above. After transfer to the experimental isolator, animals were individually housed in cages and maintained under a strictly controlled 12 light cycle, typically at sub-thermoneutral temperatures (22–26 °C). (Although thermoneutral temperatures (30–32 °C) are preferred, animal facilities are generally not engineered for thermoneutrality). The presence of bedding can confound indirect calorimetry measurements of VO_2_ and CO_2_ (e.g., through creation and release of air pockets, and contamination with gasses or particulates); therefore, we omitted bedding from the metabolic chamber when measurements were made.

At the beginning of a 24-h cycle (0600 h) of indirect open-circuit calorimetry, the calorimeter O_2_ and CO_2_ sensors were calibrated using a reference gas containing N_2_ (79%), CO_2_ (0.5%), plus O_2_ (20.5%), and the ammonia trap (Columbus Instruments) was changed. At the beginning of the nocturnal 12-h period, running wheels with associated telemetry (GP Counter Model B, Columbus Instruments) were placed inside each metabolic chamber to measure spontaneous running activity. Data, measured over 15-s intervals, are repetitively acquired and stored for each animal over 24 h (Comprehensive Lab Animal Monitoring System (CLAMS); Columbus Instruments). Spontaneous nocturnal running activity was recorded from each metabolic chamber as total running wheel revolution counts.

In our experimental design, calorimetry measurements were obtained in series, with each animal placed in an individual metabolic chamber and measurements taken in the following sequence: sample #1 air settle for 90 s; sample #1 air measure for 15 s; sample #2 air settle for 90 s; sample #2 air measure for 15 s; reference air settle for 90 s; reference air measure for 15 s; repeat the sequence over 24 h.

VO_2_, VCO_2_, and respiratory exchange ratio (RER) for each mouse in a metabolic chamber were measured every second for 15 s:

$$ {\mathrm{V}\mathrm{O}}_2={\mathrm{V}}_{\mathrm{i}}{\mathrm{O}}_{2\ \mathrm{i}}-{\mathrm{V}}_{\mathrm{o}}{\mathrm{O}}_{2\ \mathrm{o}} $$

$$ {\mathrm{V}\mathrm{CO}}_2={\mathrm{V}}_{\mathrm{o}}{\mathrm{CO}}_{2\ \mathrm{o}}-{\mathrm{V}}_{\mathrm{i}}{\mathrm{CO}}_{2\ \mathrm{i}} $$

$$ \mathrm{RER}={\mathrm{VCO}}_2/{\mathrm{VO}}_2 $$

Where:

V _i_ = Mass of air at chamber input per unit time

V _o_ = Mass of air at chamber output per unit time

O_2 i_ = Oxygen fraction in V _i_

CO_2 i_ = Carbon dioxide fraction in V _i_

O_2 o_ = Oxygen fraction in V _o_

CO_2 o_ = Carbon dioxide fraction in V _o_

Measurement errors were minimized to < 1% by (i) daily calibration of the calorimetry machine, (ii) following the manufacturer’s recommendations regarding exchanging the O_2_ and CO_2_ sensors, (iii) changing the desiccants daily, and (iv) ensuring constant flow in the inflow and outflow lines using equal length tubing, an air restrictor, and constant air pressure by the gauge on the calorimetry machine.

Body composition (lean body mass, fat mass) was measured at intervals by quantitative magnetic resonance (qMR). To perform qMR without exposing mice to environmental microbes, each animal was placed in a pre-sterilized transport device consisting of a 24-cm long by 5-cm wide glass tube with a flat bottom. A HEPA filter (ASF-4) that removes particulate material > 0.3–0.5 μm in diameter was attached to the open end of the tube and secured with a rubber band. The transport device was subsequently removed from the isolator and placed directly in the sampling port of an EchoMRI 3-in-1 Body Composition Analyzer. Following data collection (three 2-min scans/mouse), a heavy coat of 1:3:1 Clidox disinfectant was applied to the exterior of the transport device prior to its return to the isolator’s supply port. The port was then sealed and a heavy fog of atomized Clidox was introduced into the port. The transport device was retained in the supply port for a minimum of 30 min before being brought into the isolator after which time the mice were returned to their assigned cages.

## Data Availability

Bacterial V4-16S rDNA sequences in raw format (prior to post-processing and data analysis), plus sequences of ASVs, have been deposited in the European Nucleotide Archive (ENA) under study accession number PRJEB32647.

## Abbreviations

LoSF-HiFV: low saturated fat, high fruits and vegetables diet
dpg: Days post gavage
LBM: Lean body mass
PRCF: Percent relative cumulative frequency
qMR: Quantitative magnetic resonance
RER: Respiratory exchange ratio
